# Comparison of the Outcome of Patient Management with Physician Extenders Only and with both Residents and Extenders

**DOI:** 10.7759/cureus.7266

**Published:** 2020-03-14

**Authors:** Ted White, Bracken Burns, Matthew Leonard, Christian Nwabueze, Megan Quinn

**Affiliations:** 1 Trauma, Ballad Health Medical Group, Johnson City, USA; 2 Surgery, Quillen College of Medicine, East Tennessee State University, Johnson City, USA; 3 Trauma, Ballad Health Trauma Services, Johnson City, USA; 4 Epidemiology and Biostatistics, East Tennessee State University, Johnson City, USA; 5 Epidemiology and Public Health, College of Public Health, East Tennessee State University, Johnson City, USA

**Keywords:** patient outcome

## Abstract

This was a retrospective study that aimed to determine the treatment outcome of patients seen in the trauma unit of the Johnson City Medical Center (JCMC). The study included 2844 patients in the trauma registry and evaluated age, sex, injury severity score (ISS), length of stay (LOS) in the intensive care unit (ICU), overall hospital lengths of stay (LOS), ventilator days, discharge disposition, and complications between one group managed by extenders only and the second managed by both residents and extenders. The sample size of the two groups was similar (group one = 1446 and group two = 1398) and the proportions of males and females in the two groups were identical (males = 65%, females = 35%). Both groups had similar mechanisms of injury, although group one had a higher percentage of falls (32.9% vs. 22.03%) and group two had a higher proportion of motor vehicle crash (MVC) traumas (40.41% vs 30%). There was no significant difference in those discharged home and deaths between the two groups. (χ^2^(1, N = 2258) = 0.04, p = 0.82). Complications showed statistical significance when looking at extenders vs. residents plus extenders for all complications (χ^2^(7, N = 196) = 38.73, p ≤ 0.0001). It is possible that having extenders only versus both extenders and residents had no significant difference among the patient outcomes based on the variables age, sex, ISS, ICU days, overall hospital LOS, and ventilator days; however, when observing complications between the two groups, it is possible that patients are more likely to have complications due to overall hospital LOS in the residents plus extenders group.

## Introduction

Physician extenders are healthcare providers who are not considered physicians but who perform medical activities typically performed by a physician, most commonly a nurse practitioner or a physician assistant [1]. Extenders have shown to significantly reduce the length of stay (LOS) in a pediatric chronic ventilator-dependent unit, statistically significant reductions in floor and intensive care unit (ICU), and overall hospital LOS [2-4]. Critical care mortality has also been observed with the inclusion of extenders in ICUs. One study found that patients in ICUs with nurse practitioners and physician assistants had lower mean acute physiology scores and mechanical ventilation rates [5]. Another study examining the use of nurse practitioners and physician assistants on adult surgical and trauma services also found that LOS decreased but found that mortality and morbidity were unchanged [6]. With some research showing that extenders had a positive effect when introduced in certain hospital settings, it is worthwhile to determine if there a significant treatment outcome between physician extenders only and both residents and extenders. The current study aimed to determine the treatment outcome of patients seen in the Trauma unit of Johnson City Medical Center (JCMC). There were two groups of patients; those managed by physician extenders only (those seen between July 1, 2013 and September 30, 2014) and those managed by both general surgery residents and physician extenders (those seen between October 1, 2014 and December 31, 2015). The staffing coverage provided by the physician extender-only group consisted of three extenders during the daytime hours and one extender during night-time hours. The staffing coverage provided by the group of general surgery residents and physician extenders included two extenders and a second-year general surgery resident in their critical care rotation during the daytime hours and residents only at night including an intern and a third-year resident. Both groups had in-house trauma attending coverage at night. The aim of the current analysis was to determine if there were differences in age, mechanism of injury, LOS in the ICU, and outcome of the admission between the two staffing models.

## Materials and methods

This study received an exemption from the East Tennessee State University Institutional Review Board (c0816.20e). The study included 2844 patients in the trauma registry. The design was cross-sectional and analyzed the association between differences in age, mechanism of injuries, LOS in the ICU, and outcome of the admission between the two groups. Correlation coefficients were determined for the associations between differences in age, mechanism of injuries, and LOS in the ICU. T-tests were used to determine differences in mean length of hospital stay (LOHS), injury severity score (ISS), and LOS in the ICU between the two groups. For disposition of patients for the two groups and for the comparison of complications, a chi-squared test was used. The chi-square test was done by means of including all the complications from both groups and comparing them between the two. Finally, a comparison of complications between the two groups was also done using multiple logistic regression while controlling for the variables of age, sex, ISS, ICU days, LOS, and ventilator days to see if study group one or two was more likely to have complications.

## Results

Study groups

Study Group 1 (July 1, 2013 to September 30, 2014)

The first group (2013/2014 treatment group) managed by extenders only consisted of 1446 patients admitted into the trauma unit of JCMC between July 1, 2013 and September 30, 2014. Thirty-five percent (N = 501) were females, while 65% (N = 945) were males. Their age ranged from < 1 year to 97.3 years with a mean age of 44 years. The mechanisms of injuries were varied and included aircraft, lawn mowers, skating, burns, and many other mechanisms, but the most common mechanisms of injuries were falls (N = 476, 32.9%), motor vehicle crash (MVC; N = 437, 30%) and motorcycle accidents (N = 108, 7.4%). The ISS ranged from zero to 75 with a mean score of 11; the range of total hospital stay was from 1 day to 97 days with a mean period of 4.3 days, while the mean length of ICU stay was 1 day but ranged from 1 day to 49 days. Throughout the period under review as seen in Table 1, there were 51 deaths (3.52%); 133 (9.91%) people went to rehabilitation and 1059 (73.23%) people were discharged home. Of the other patients, they either left against medical advice (AMA), were sent to nursing homes, jail, psychiatric hospitals, or skilled nursing facility (SNF). Common complications during hospital stay during this period included seven cases of deep venous thrombosis (DVT), nine cases of pneumonia, five of unintended extubation, nine cases of unplanned intubation, 10 cases of readmission, and two cases of acute respiratory distress syndrome. There was one case of aspiration pneumonia but no case of acute renal failure.

**Table 1:** Treatment outcomes of trauma patients at JCMC (managed by extenders only) AMA, against medical advice; SNF, skilled nursing facility

Outcome	Frequency	Percentage	Cumulative %
Death	51	3.53	3.52
Rehabilitation	133	9.19	12.71
Home	1059	73.24	85.94
AMA, nursing home, jail, psychiatric hospitals, SNF	203	14.04	100

There was no correlation between age and length of ICU stay (correlation coefficient = 0.024, p = 0.350), a weak positive correlation between age and the length of hospital stay (correlation coefficient = 0.129, p ≤ 0.0001), and a strong positive correlation between length of hospital stay and length of ICU stay (correlation coefficient = 0.698, p ≤ 0.0001). There was weak positive correlation between ISS and age (correlation coefficient = 0.108, p ≤ 0.0001,) between ISS and length of hospital stay (correlation coefficient = 0.371, p ≤ 0.0001) and ISS and length of ICU stay (correlation coefficient = 0.358, p ≤ 0.0001). *Study Group Two (Oct 1, 2014 to Dec 31, 2015)* The second group who was managed by residents and extenders consisted of 1398 patients admitted into the trauma unit of JCMC between October 1, 2014 and December 31, 2015. Thirty-five percent (N = 490) were females, while 65% (N = 908) were males. The mean age was 40 years with a range from <1 year to 95.4 years. The most common mechanisms of injury were MVC (N = 565, 40.41%), falls (N = 308, 22.03%), and motorcycle accidents (N = 104, 7.43%). The ISS ranged from zero to 75 with a mean score of 10, and the range of total hospital stay was from 1 day to 67 days with a mean period of 4.4 days, while the mean length of ICU stay was 1.1 days but ranged from 0 day to 59 days. As seen in Table 2, there were 55 deaths (3.93%), 95 (6.60%) went to rehabilitation, and 1093 (78.18%) people were discharged home, while others were sent to either nursing homes, jails, psychiatric hospitals, or other facilities. Common complications during hospital stay during this period include 14 cases of deep venous thrombosis (DVT), 25 cases of pneumonia, five cases of unintended extubation, seven cases of unplanned intubation, five readmission, and no cases of acute respiratory distress syndrome or aspiration pneumonia but one case of acute renal failure. 

**Table 2:** Treatment outcomes of trauma patients at JCMC (managed by both residents and extenders) AMA, against medical advice; SNF, skilled nursing facility

Outcome	Frequency	Percentage	Cumulative %
Death	55	3.93	3.93
Rehabilitation	95	6.79	10.53
Home	1093	78.18	88.71
AMA, nursing home, jail, psychiatric hospitals, SNF	155	11.09	100

**Figure 1:**
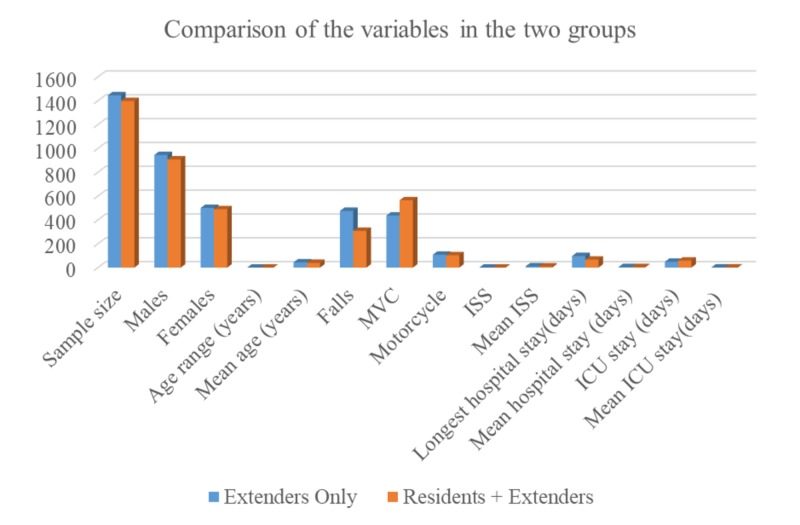
Comparison of the variables in the two groups

There was no correlation between age and length of ICU stay (correlation coefficient = 0.073, p = 0.0002), a weak positive correlation between age and the length of hospital stay (correlation coefficient = 0.203, p ≤ 0.0001), and a strong positive correlation between length of hospital stay and length of ICU stay (correlation coefficient = 0.673, p ≤ 0.0001). There was weak positive correlation between ISS and age (correlation coefficient = 0.224, p ≤ 0.0001,) between ISS and length of hospital stay (correlation coefficient = 0.456, p ≤ 0.0001), and ISS and length of ICU stay (correlation coefficient = 0.389, p ≤ 0.0001). *Comparison of the Outcomes of Groups One and Two* Figure 1 shows a visual comparison of the variables between the two groups. Group one consisted of patients who were managed by extenders alone, while group two consisted of patients managed by both the extenders and resident doctors. The sample size of the two groups was similar (group one = 1446 and group two = 1398), and the proportions of males and females in the two groups were identical (males = 65%, females = 35%). The age range and mean age (group one = 0-97 years, 44 years and group two = 0-95 years, 40 years) were similar in the two groups. There was a higher percentage of falls in group one than in group two (32.9% vs 22.03%), but a higher proportion of MVC traumas in group two than group one (40.41% vs 30%). The percentage of trauma from the motorcycle was similar in both groups. Table 3 illustrates the differences in mean for ISS, LOHS, and ICU LOS, between groups one and two.

**Table 3:** Comparison of the study group 1 and study group 2 ICU, intensive care unit

	Group 1		Group 2		
Factors	Frequency	Percent	Frequency	Percent	P-Value
Sample size	1446		1398		
Males	945	65	908	65	
Females	501	35	490	35	
Age range	0-97		0-95		
Mean age	44		40		
Injury mechanism					
Falls	476	32.9	308	22.03	
MVC	437	30	565	40.41	
Motorcycles	108	7.4	104	7.43	
ISS	0-75		0-75		
Mean ISS	11		10		0.009
Longest ICU stay (days)	49		59		
Mean ICU stay (days)	1		1.1		0.119
Longest hospital stay (days)	97		67		
Mean hospital stay (days)	4.3		4.4		0.584
Longest vent days	59		66		
Mean vent days	0.5		0.5		0.856

Seventy-one percent of group one population and 76% of the group two population were discharged home, while 3.4% and 3.82% of groups one and two, respectively, died while on admission (Table 4). Looking at the disposition, there was no significant difference in those discharged home and deaths between the two groups. (x2(1, N = 2258) = 0.04, p = 0.82.) 

**Table 4:** Disposition of hospital stay

	Group 1	Group 2	
Disposition	Frequency	Frequency	Total
Discharged home	1059	1093	2152
Deaths	51	55	106
Total	1110	1148	2258

Both groups had their lowest ISS score, being 0 and highest 75. The mean ISS score was 11 vs. 10. The LOHS was longer in the first group (97 days vs. 67 days), but the mean LOHS was similar (4.4 days vs. 4.5 days). However, the second group had a longer length of ICU stay than the first group (59 days vs. 49 days), but the average length of ICU stay was similar in both groups (1.2 days vs. 1.0 day). Group two also had the highest ventilator days with 66 vs. 59 in group one. Looking at the means of the four groups, ISS showed a significance between the means (t (2842) = 2.588, p = 0.009). There was 0.547, (p = 0.584), the mean length of ICU stay (t (2819) = -1.559, p = 0.119), and ventilator days (t (2806) = 0.1807, p = 0.85659). With reference to the complications, there were more cases of DVT, pneumonia, and acute renal failure in group two than in group one (Table 5). On the other hand, there were more cases of unplanned intubation, re-admission, adult respiratory distress syndrome (ARDS), and aspiration pneumonia in group one as compared to group two. Looking at these data tests showed that the numbers were greatly significant, suggesting that complications could be dependent on the two groups when looking at extenders vs. residents plus extenders for all complications as a whole (χ^2^ (7, N = 196) = 38.73, p ≤ 0.0001).

**Table 5:** Comparison of the complications in the study groups 1 & 2 ARDS, acute respiratory distress syndrome

	Group 1	Group 2
DVT	7	14
Pneumonia	9	25
Unintended extubation	5	5
Unintended intubation	9	7
Re-admission	10	5
ARDS	2	0
Acute renal failure	0	1
Aspiration pneumonia	1	0

*Logistic Regression of the Two Groups* Study group one: The results of the logistic regression analysis for the study group one show that the full model which considered all the six independent variables together was statistically significant (P ≤ 0.0001). This predicted the odds of a patient having complications (yes or no) and included the following independent variables age, sex, ISS, ICU days, LOHS, and vent days. Wald statistics indicate that ISS, ICU days, and LOHS stay predict complications and are statistically significant. The odds of complications are 4% more likely for every 1 unit increase in ISS, while other variables in the model were held constant (OR = 1.038, P ≤ 0.0001, confidence interval = 1.019-1.057). The odds of complications are 20% more likely for every 1-day increase in ICU days, while other variables in the model were held constant (OR = 1.202, P ≤ 0.0001, CI = 1.097-1.318). The odds of complications are 6% more likely for every 1-day increase in LOHS, while other variables in the model are held constant (OR = 1.060, P = 0.002, CI = 1.021-1.101). Age showed no significance associated with complications (OR = 1.008, P = 0.110, CI = 0.998-1.019). The odds of complications were 5% more likely in males compared to females if other variables in the model were held constant; however, this was not a statistically significant difference (OR = 1.050, P = 0.855, CI = 0.617-1.788). The odds of complications were 6% less likely for every 1-day increase in vent days, while other variables in the model were held constant, but this was not a statistically significant difference (OR = 0.948, P = 0.157, CI = 0.880-1.020). 

The logistic regression model used was tested for goodness of fit by the Hosmer and Lemeshow test, which shows that the model used fits the study with a significant value of 0.99(>0.05). Study group two: The results of the logistic regression analysis for study group two show that the full model which considered all six independent variables together was statistically significant (P ≤ 0.0001). This predicted the odds of a patient having complications and included the following independent variables age, sex, ISS, ICU days, LOHS, and ventilator days. Wald statistics indicate that ISS, ICU days, and LOHS stay predict complications and are statistically significant. The odds of complications were 3% more likely for every 1 unit increase in ISS while other variables in the model were held constant (OR = 1.037, P = 0.001, CI = 1.014-1.060). The odds of complications were 18% more likely for every 1-day increase in ICU days, while other variables in the model were held constant (OR = 1.182, P = 0.0004, CI = 1.078-1.297). The odds of complications were 14% more likely for every 1-day increase in LOHS days, while other variables in the model were held constant (OR = 1.145, P ≤ 0.0001, CI = 1.106-1.18). Age showed no significance associated with complications (OR = 1.006, P = 0.224, CI = 0.995-1.018). The odds of complications were 5% more likely in males compared to females if other variables in the model were held constant; however, this was not statistically significant (OR=0.644, P=0.103), CI= 0.379-1.093. The odds of complications were 2% more likely for every 1-day increase in Vent days (OR=1.016, P=0.778), CI= 0.906-1.140, but this was not a statistically significant difference. The logistic regression model used was tested for goodness of fit by Hosmer and Lemeshow test which shows that the model used fits the study with a significant value of 0.98(>0.05). 

## Discussion

This study aimed to compare the treatment outcome in patients treated in the trauma unit at JCMC. Observation of the two groups, extenders (group one) and extender plus residents (group two), demonstrated that both study groups showed a moderate to strong correlation between the LOHS and ICU stay, suggesting that the two may be associated with one another, which was expected. It was also observed among the two groups that age and ISS had a weak correlation while ISS and LOHS, and ISS and length of ICU stay showed weak to moderate correlation. Furthermore, there was no significant difference between the mean of hospital and ICU stay suggesting that extenders or extenders plus residents had no effect on the average LOS. 

There is little known of the impact of extenders and/or residents and the outcome of patient management in patients admitted through the trauma unit. A study completed by Timmermans et al. demonstrated that there was no difference in LOS, quality of care and mortality in general medical patients under the care of physician assistants (PAs) in collaboration with medical doctors (MDs) and patients under the care of MDs only [7]. This directly reflects our results in those patients who were admitted through the trauma unit. Another study showed contradicting results showing that there was an increased LOS in general medical patients under the care of hospitalists/PA compared to the resident based team, but similar mortality rates. The hospitalists/PA group had a higher percentage of patients transferred to the ICU after admission compared to the resident based team, which could be cause for longer LOS [8].

When directly comparing the variables between groups, the data showed close similarities between the variables of the two groups except for the frequency of falls and motor vehicle accidents. This seemed to have little impact on the outcome of the patient. There was no significant difference between the two groups and the disposition of patients in those being discharged home versus death. In regards to complications, there were significant differences between the two groups. Less complications occurred in the extenders-only group compared to extenders plus residents. Russell *et al*. demonstrated that neuroscience patients managed by an acute care nurse practitioner (ACNP) had fewer complications such as urinary tract infections, skin breakdown, and pneumonia compared to those patients not managed by an ACNP [9]. Finally, we found that ISS, ICU days, and LOHS in both groups were significant while the other variables observed were not. The odds ratios of the three significant variables ISS, ICU days, and LOHS were similar, but LOHS in study group two was slightly higher than in study group one. This suggests that complications for extenders only versus residents plus extenders have no difference except for maybe LOHS. 

## Conclusions

It is possible that having extenders only versus both extenders and residents had no significant difference in the length of time in hospital and length of time in the ICU, age and ISS score, ISS score and LOHS, and ISS score and LOS in the ICU despite a weak to strong correlation among these variables. Length of hospital stay in study group two (residents plus extenders) had a slightly higher significance among complications between extenders only versus residents plus extenders while the other variables were either not significant or significant but of similar value between the two study groups. It is possible that patients are more likely to have complications due to LOHS in the residents plus the extenders group. Further research is needed to better understand the relationship between staffing models and patient complications.
